# Early taxane exposure and neurotoxicity in breast cancer patients

**DOI:** 10.1007/s00520-024-08908-2

**Published:** 2024-10-08

**Authors:** Erika Cimbro, Mariele Dessì, Pina Ziranu, Clelia Madeddu, Francesco Atzori, Eleonora Lai, Andrea Pretta, Stefano Mariani, Clelia Donisi, Dario Spanu, Marta Pozzari, Sara Murgia, Giorgio Saba, Claudia Codipietro, Enrico Palmas, Giorgia Sanna, Francesca Semonella, Salvatore Sardo, Gabriele Finco, Mario Scartozzi

**Affiliations:** 1 Medical Oncology Unit, University Hospital and University of Cagliari, 09042 Cagliari, Italy; 2 Department of Medical Sciences and Public Health, University of Cagliari, 09042 Cagliari, Italy; 3 Department of Anesthesia, Resuscitation and Pain Therapy, University of Cagliari, 09042 Cagliari, Italy

**Keywords:** Taxane, Neurotoxicity, Quality of life

## Abstract

**Introduction:**

Breast cancer is the most diagnosed tumor and a leading cause of cancer death in women worldwide. Taxanes are the most used chemotherapeutic agents and are strictly connected to neurotoxicity. Taxane-induced neuropathy (TIN) significantly impacts patients’ quality of life (QOL). Early identification and management of TIN could improve preventive strategies to preserve patients’ QOL during and after breast cancer treatment.

**Objective:**

This prospective, observational study aimed to evaluate the taxane-induced neuropathy (TIN) in early breast cancer patients treated with weekly paclitaxel at an earlier stage and identify any correlation between TIN and QOL.

**Methods:**

Data from stage I-III breast cancer patients treated with taxane-based therapy between 2018 and 2022 were collected at the Medical Oncology Unit of the University Hospital of Cagliari. Peripheral neuropathy was evaluated using the NCI-CTCAE scale (National Cancer Institute, Common Terminology Criteria for Adverse Events) at every drug administration. In contrast, QOL was assessed using EORTC QLC-CIPN20 and FACT-Taxane questionnaire at baseline (T0), after 4 weeks (T1) and 12 (T2) weeks of treatment. Statistical analysis was performed to evaluate the correlation between neurotoxicity and QOL.

**Results:**

Neurotoxicity incidence peaked at the third, fourth, and sixth week of treatment, with patients reporting grade 1 and 2 neurotoxicity. Simultaneously with increasing doses of paclitaxel, significant differences in QOL were observed in early treatment cycles relating to TIN presentation. Patients with higher neurotoxicity grades reported lower QOL scores.

**Conclusions:**

Despite the absence of effective treatments to prevent paclitaxel-induced neurotoxicity, symptoms are managed through dosage reduction, delay, or treatment interruption. Future research should focus on identifying neuroprotective measures to avoid an irreversible decline in the quality of life for breast cancer survivors.

## Introduction

Breast cancer is the most diagnosed and prevalent cancer and a leading cause of cancer death in women worldwide [1]. According to the recent 2020 data report, breast cancer accounts for 2,261,419 cases in the world [2]. Despite the development of new antineoplastic drugs, used alone or in combination, chemotherapy represents the first choice of treatment in neoadjuvant or adjuvant settings, leading to an enhanced disease-free survival rate [3, 4]. The multidisciplinary approach is crucial to maximize therapeutic efficacy while reducing drug adverse reactions [5]. Yet, the polychemotherapy approach shows relatively high toxicity, profoundly affecting the quality of life (QOL)of the patients. Over 90% of them reported one or more symptoms directly caused by chemotherapy [6]. One of the chemotherapeutic agents used is taxane, which is known to induce peripheral neuropathy. Based on the timing of onset, neuropathy can be acute (occurring within 24 h after the administration of the antineoplastic agent) with an incidence of 97% or chronic (a long-lasting effect that happens even after the end of the treatment and persists for months or years) with an incidence of 64%. Taxane-induced neuropathy (TIN) is often irreversible and can persist for many years after chemotherapy, sometimes indefinitely [7, 8]. Based on signs and severity, neurotoxic manifestations of anticancer agents can be divided into five categories: sensory neuropathy, motor neuropathy, autonomic neuropathy, myopathy or myopathic effects, and central neuropathy. Taxanes impact all sensory neurons, particularly thick myelinated nerve fibers responsible for vibration, sensation, and proprioception [9]. The related symptoms include paresthesia, dysesthesia, numbness, burning and shooting or electric shock sensation, hyperalgesia, and allodynia, often in a “stocking and glove” distribution [10]. The degree of TIN depends on variable factors such as the cumulative dose of chemotherapy, the use of other concomitant neurotoxic chemotherapy (i.e., carboplatin), the time of exposure to the drug, and the therapy duration [11, 12]. With further administration increases, symptoms can evolve, resulting in loss of movements and reflexes.

TIN can significantly impact a patient’s QOL, especially for long-term survivors of early breast cancer.

Previous research on several cancer types has shown a negative association between chemotherapy-induced peripheral neuropathy (CIPN) burden and health-related quality of life (HRQOL) in both cancer survivors and patients with advanced cancer [13]. However, until now, there have been few systematic assessments of the impact of peripheral neuropathy problems on QOL, which the lack of adequate measures may partly explain. In attempting to address this issue, specific neurotoxicity evaluation tools have been added to QOL measures. Recently, in a review on the incidence and impact of persistent taxane-induced peripheral neuropathy, Hertz demonstrated that persistent TIN adversely affects long-term functional abilities, such as gait, balance, and the ability to work, but also reduces overall QOL, particularly impacting physical and social aspects [14].

Proactively addressing TIN through early detection not only improves the quality of life for early breast cancer survivors but also opens the door to more effective treatments and preventive measures against this debilitating condition [7, 8].

### Aim of the study

This study aimed to evaluate TIN in early breast cancer patients treated with weekly paclitaxel in an earlier stage and identify a correlation between TIN and QOL.

## Materials and methods

The study assessed all consecutive patients with non-metastatic (I-III) breast cancer who underwent taxane-based treatment between 2018 and 2022 at the Medical Oncology Unit of the University Hospital of Cagliari. The patients enrolled received a chemotherapy regimen consisting of anthracycline (epirubicin 90 mg/m^2^, cyclophosphamide 600 mg/m^2^) for four cycles, followed by weekly paclitaxel (80 mg/m^2^ for 12 weeks) in either a neoadjuvant or adjuvant setting. Eligible patients were over 18 years old, with ECOG performance status ≤ 2 without significant organ dysfunction, a diagnosis of stage I-III breast cancer.

The study protocol was approved by the Institutional Ethics Committee of the “Azienda Ospedaliero Universitaria” of Cagliari. It was conducted in accordance with the guidelines of the Declaration of Helsinki and Good Clinical Practice. Written informed consent was obtained from all patients.

### Assessment of neurotoxicity and quality of life questionnaires

The following questionnaires were used to assess neurotoxicity and QOL:

- NCI-CTCAE (National Cancer Institute, Common Terminology Criteria for Adverse Events) is a descriptive terminology for reporting adverse events. Each unfavorable event is assigned a severity scale. For peripheral sensory and motor neuropathy, G1 is asymptomatic, G2 includes mild symptoms that limit instrumental activities of daily living (ADL), and G3 includes severe symptoms that limit self-care ADL. For paraesthesia, G1 includes mild symptoms, G2 moderate symptoms limiting instrumental ADL, and G3 severe symptoms limiting self-care ADL. G4 and G5 represent more critical conditions that require urgent intervention or pose a risk of death.
- EORTC QLQ-CIPN20 (European Organization for Research and Treatment of Cancer Quality of Life Questionnaire, Chemotherapy Induced Peripheral Neuropathy) assesses the symptoms and side effects of chemotherapy-induced peripheral neuropathy (CIPN), indicating the severity and impact of peripheral neuropathy on the patient’s quality of life (https://www.eortc.org/app/uploads/sites/2/2018/08/Specimen-CIPN20-English.pdf) . It consists of 20 items, each rated from 1 (not at all) to 4 (very much). The average score is standardized to a 0–100 range, with higher scores indicating worse symptoms.
- FACT-Taxane (Functional Assessment of Cancer Therapy, Taxane, version 4) is a patient-reported measure of QOL for patients receiving taxane-based treatments. It contains 43 items distributed across five subscales: Physical Well-Being (PWB, seven items), Social/Family Well Being (SWB, seven items), Emotional Well-Being (EWB, six items), Functional Well Being (FWB, seven items), and additional concerns regarding Taxane Therapy (Tax-Subscale, 16 items). Each item is rated on a scale from 0 (not at all) to 4 (very much), with some items scored in reverse according to the scoring guidelines. The subscale scores are summed to provide an overall quality of life score ranging from 0 to 172, with higher scores indicating better QOL (https://www.facit.org/_files/ugd/626819_f902fdea55424b4aa7f5aa19a51ed08c.pdf) .

### Data collection

An oncologist assessed peripheral neuropathy in all enrolled patients weekly using the CTCAE scale before each drug administration, at baseline, after 1 week (cycle I), and beyond.

To accurately quantify neurotoxicity, the EORTC QLQ-CIPN20 scale was used. Additionally, to evaluate the impact of peripheral neuropathy on QOL, the FACT-Taxane questionnaire was administered. Both assessments were conducted after 1 week (T0), 4 weeks (T1), and 12 weeks (T2) of treatment. When necessary, staff provided neutral assistance for survey completion and prompted patients to fulfil incomplete items. If the patient refused, it was indicated on the questionnaire. If a patient missed a scheduled appointment, the questionnaire was completed by telephone on the appointed date or at the rescheduled appointment.

### Outcomes

We focused on the symptoms induced by taxane-related neurotoxicity, assessing its presentation and development through the CTCAE score, the EORTC QLQ-CIPN20, and the Tax-Subscale score. We evaluated the association between the CTCAE grade and the questionnaire scores at each time of taxane administration. Moreover, we investigated the evolution of the FACT-Taxane score for each patient at different times and its correlation with the EORTC QLQ-CIPN20 score.

### Statistical analysis

Statistical analysis was performed with the MedCalc Statistical Software Version 14.10.2 (MedCalc Software bvba, Ostend, Belgium; http://www.medcalc.org; 2014). The difference between different timings was assessed using an ANOVA test for repeated measures using Bonferroni’s correction for multiple comparisons. We used the Kruskal–Wallis test to determine the association between neurotoxicity grade and quality of life. To assess the strength of the correlation between the different QOL questionnaires, we used Spearmann’s Rho test. All analyses were performed using two-sided tests with a 5% type-I error rate. Statistical significance was set at a two-tailed *p*-value of < 0.05.

## Results

Between January 2018 and December 2022, 300 patients were enrolled. Baseline characteristics are reported in Table 1. Most of the patients presented ECOG PS 1 (77.3%) or 0 (22.7%); half of them (49.3%) presented comorbidities such as hypertension (23.3%), diabetes (13.7%), depression (8.3%), and impaired thyroid function (18%). None of these patients presented peripheral neuropathy at the time of enrollment. Of these, 261 patients completed the study, while 39 discontinued early for the following reasons: 5 patients passed away during treatment, 14 completed their treatment at different centers, and 20 did not finish the treatment due to logistical problems or by choice and were considered lost to follow-up. All patients included in the analysis were assessed by a physician using CTCAE and filled out the questionnaires every week.

**Table 1:** Patient demographics and disease characteristics at baseline

Characteristics	Total	(*N* = 300)
Age, y, median ± SD (range)	56.2 ± 11.0	(32–85)
Alive	295	98.3%
ECOG PS score		
0	68	22.7%
1	232	77.3%
2	None	
Comorbidities	148	49.3%
Diabetes	41	13.7%
Hypertension	70	23.3%
Depression	25	8.3%
Thyroid disfunction	54	18%
Breast cancer type		
Invasive ductal carcinoma	281	93.7%
Invasive lobular carcinoma	14	4.7%
Others	5	1.6%
Stage		
I	81	27%
II	161	53.6%
III	58	19.4%
Regimen		
Adjuvant	236	78.7%
Neoadjuvant	64	21.3%
CTCAE score		
G0	300	
G1	None	
G2	None	
G3	None	

### Neurotoxicity assessment

**Fig. 1:**
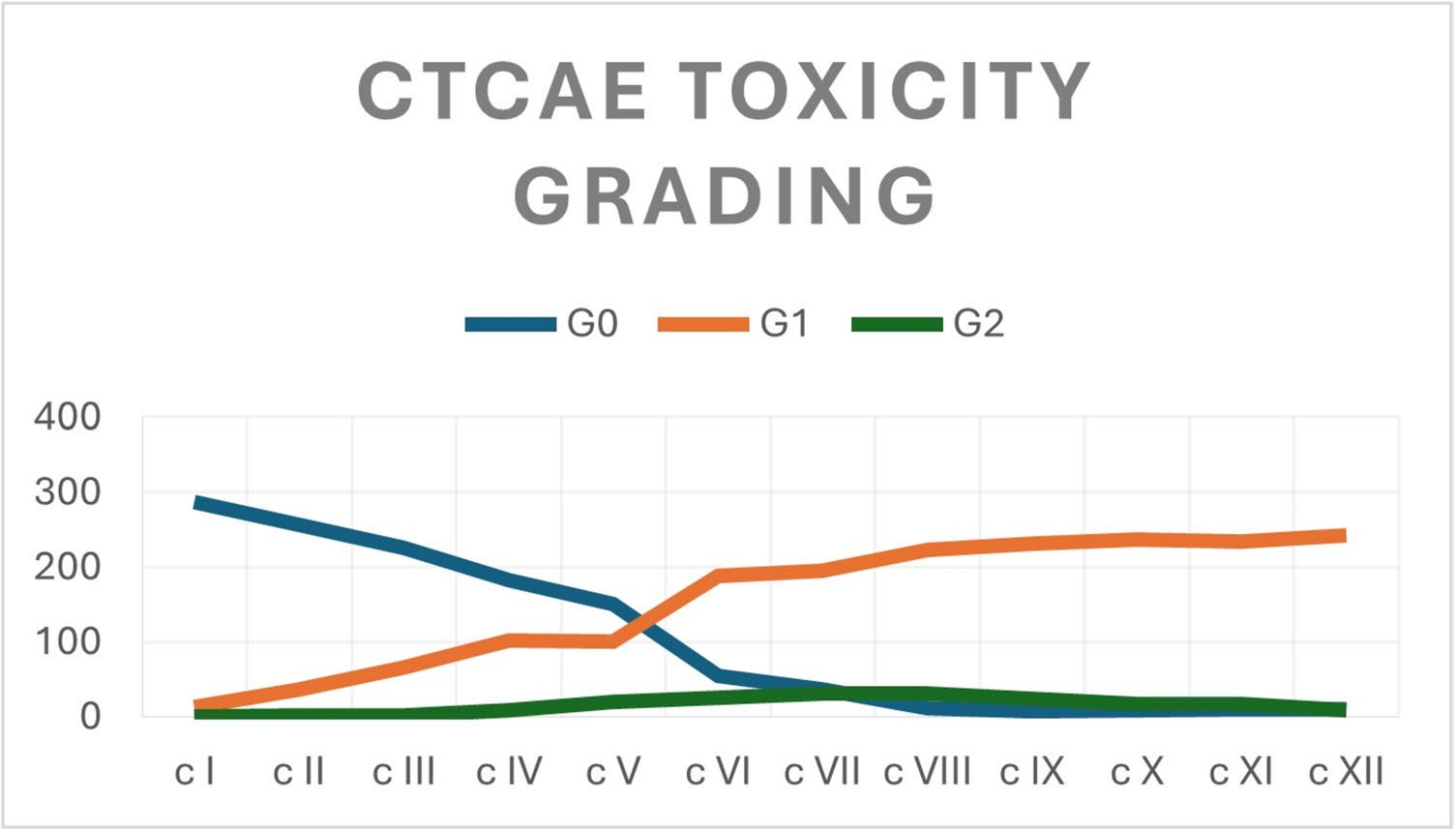
Total of patients and corresponding CTCAE grades at each cycle

Neurotoxicity grades assessed via CTCAE score are shown in Table 2 and Fig. 1.

**Table 2:** CTCAE grade of Toxicity at different administrations and relative incidence

CTCAE grade	c I	c II	c III	c IV	c V	c VI	c VII	c VIII	c IX	c X	c XI	c XII
	(No. 300)	(No. 296)	(No. 294)	(No. 294)	(No. 272)	(No. 269)	(No. 265)	(No. 265)	(No. 264)	(No. 263)	(No. 261)	(No. 261)
G0 No. (%)	287 (95.7)	257 (86.8)	226 (76.9)	183 (62.2)	151 (55.5)	55 (20.4)	38 (14.3)	11 (4.1)	8 (3)	9 (3.4)	10 (3.8)	10 (3.8)
G1 No. (%)	13 (4.3)	37 (12.5)	66 (22.4)	102 (34.7)	101 (37.1)	189 (70.3)	196 (74.0)	223 (84.2)	232 (87.9)	237 (90.1)	234 (89.7)	242 (92.7)
G2 No. (%)	0	2 (0.7)	2 (0.7)	9 (3.1)	20 (7.4)	25 (9.3)	31 (11.7)	31 (11.7)	24 (9.1)	17 (6.5)	17 (6.5)	9 (3.4)

Notably, early toxicity was observed in 34.7% of patients at T1 after a cumulative dose of 320 mg/m^2^ and in 92.7% at T2 when the cumulative dose reached 960 mg/m^2^.

The association between the CTCAE score, EORTC QLQ-CIPN20, and Tax-Subscale scores was assessed at different timings: T0 (cycle I), T1 (cycle IV), and T2 (cycle XII). We found that patients with a higher grade of CTCAE toxicity reported a significantly higher score of EORTC QLQ-CIPN20 in comparison to patients with a lower grade of CTCAE score at cycles I, IV, and XII (*p* < 0.001).

In detail, at cycle I, patients with G0 neurotoxicity achieved a median value of 3.5 in the EORTC QLQ-CIPN20, compared to a median value of 15.8 in patients with G1 toxicity. No patients reported G2 neurotoxicity on the CTCAE scale.

At cycle IV, patients without neurotoxicity achieved a median value of 21 in the EORTC QLQ-CIPN20, compared to 24.5 in the patients with G1 toxicity and 42.1 in the group of patients with G2 toxicity.

**Fig. 2:**
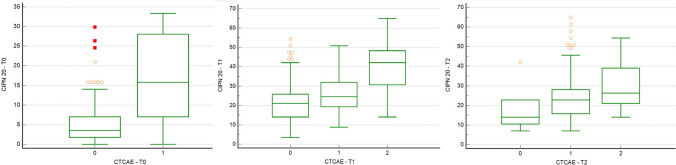
Neurotoxicity and the EORTC QLQ-CIPN20 at cycle I (T0), IV (T1), and XII (T2)

Similarly, at cycle XII, patients without neurotoxicity achieved a median value of 14 on the EORTC QLQ-CIPN20, compared to 22.8 in patients with grade 1 toxicity and 26.3 in the group of patients with grade 2 toxicity (Fig. 2).

A significant difference was found in neurotoxicity assessed via the Tax-Subscale at cycle I (*p* < 0.001), cycle IV (*p* < 0.001), and cycle XII (*p* < 0.001).

At cycle I, patients without neurotoxicity achieved a median value of 1 in the Tax-Subscale, compared to 18.2 in the patients with G1 toxicity. No patients reported G2 neurotoxicity.

At cycle IV, patients without neurotoxicity achieved a median value of 13.8 in the Tax-Subscale questionnaire, compared to 15.7 in the patients with G1 toxicity and 26.9 in the group of patients with G2 toxicity.

**Fig. 3:**
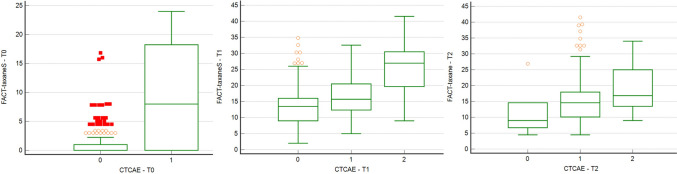
Neurotoxicity and the FACT-Taxane Tax-Subscale at cycle I–IV–XII

Similarly, at cycle XII, patients without neurotoxicity achieved a median value of 8.9 on the Tax-Subscale, compared to a median value of 14.6 in the patients with grade 1 toxicity and 16.8 in the group of patients with grade 2 toxicity (Fig. 3).

### FACT-Taxane questionnaire

Repeated measures ANOVA confirmed a statically significant difference in the FACT-Taxane questionnaire at T0 in comparison to the IV cycle (*p* < 0.001) and XII cycle (*p* < 0.001), showing worsening of condition with a mean value of 131.5, 114.3, and 113.5, respectively. No significant statistical difference is shown between the IV and XII cycles (Table 3).

**Table 3:** Significant difference for FACT-Taxane questionnaire assessed through RM ANOVA

Post hoc comparisons—FACT-Taxane						
		Mean difference	SE	*t*	*p*_bonf_	*p*_holm_
t0	t1	17.216	0.452	38.104	< 0.001	< 0.001
	t2	17.978	0.452	39.791	< 0.001	< 0.001
t1	t2	0.762	0.452	1.687	0.277	0.092

Data demonstrate a concordance between the CTCAE scale and the quality of life established through the FACT-Taxane scale at cycle I (*p* < 0.001) and cycle IV (*p* < 0.001) but not at cycle XII (*p* = 0.3). At cycle I, patients without neurotoxicity achieved a median value of 132.3 in the FACT-Taxane questionnaire, compared to a median value of 110.3 in the patients with grade 1 toxicity. No patients reported G2 neurotoxicity.

At cycle IV, patients without neurotoxicity achieved a median value of 116.9 in the FACT-Taxane questionnaire, compared to 114.8 in the patients with G1 toxicity and 101 in the group of patients with G2 toxicity.

**Fig. 4:**
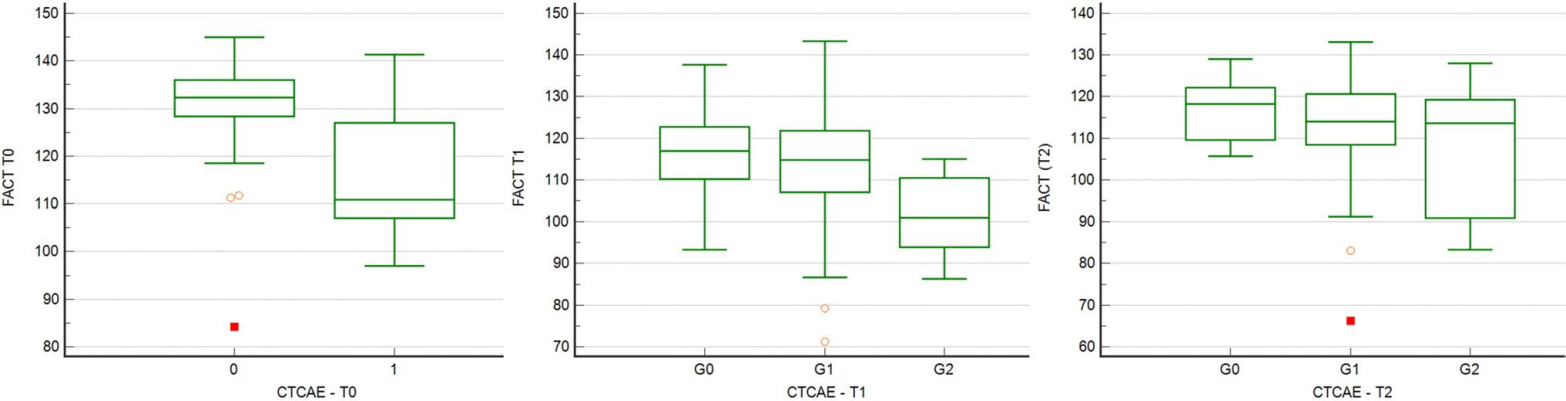
Neurotoxicity and the FACT-Taxane at cycle I–IV–XII

Conversely, at cycle XII, patients without neurotoxicity achieved a median value of 118.2 on the FACT-Taxane questionnaire, compared to a median of 114 in the patients with G1 toxicity and 113.6 in the group of patients with G2 toxicity (Fig. 4). This trend does not reach statistical significance.

### Correlation between FACT-Taxane and EORTC QLQ-CIPN20

Our results suggest a possible correlation between FACT-Taxane and EORTC QLQ-CIPN20. Our analysis found a statistically significant correlation between the two questionnaires assessed at multiple time points. Among the subscales, we found a significant correlation between the EORTC QLQ-CIPN20 and Tax-Subscale at cycle IV (*p* < 0.0001) and cycle XII (*p* < 0.0001). No significant correlation with the other subscales was found.

## Discussion

The study found that taxane-induced neuropathy (TIN) in breast cancer patients increased with higher cumulative doses of paclitaxel. Early toxicity occurred in 34.7% of patients at 320 mg/m^2^ and 92.7% at 960 mg/m^2^. Higher neurotoxicity was linked to worse quality of life (QOL) scores, with the EORTC QLQ-CIPN20 and FACT-Taxane questionnaires showing similar effectiveness in assessing neurotoxicity.

We observed a rate of TIN during taxane chemotherapy in patients with non-metastatic breast cancer in the neoadjuvant and adjuvant settings similar to those reported in the literature in the same setting [15–17].

Our results are consistent with the evidence that the rate of peripheral neuropathy with taxanes correlates with cumulative dose delivered and dose per treatment cycle. Indeed, we found that 34.7% of patients experienced early toxicity when they were treated with weekly paclitaxel at a cumulative dose of 320 mg/m^2^ (T1). Furthermore, as the cumulative dose of paclitaxel reached 960 mg/m^2^ (T2), the percentage of patients experiencing early toxicity increased to 92.7%.

Van Gerven et al. found that CIPN from paclitaxel is typically mild or subclinical up to a cumulative dose of 1400 mg/m^2^ when administered at 135 or 175 mg/m^2^ every 21 days (Q21) [18]. However, data regarding a threshold for neuropathy onset are controversial. Furthermore, in a randomized phase III study of metastatic breast cancer, Jones found that the average cumulative dose of paclitaxel leading to the onset of grade 2 peripheral neuropathy was 715 mg/m^2^ [19].

The present study found a significant association between the development of TIN assessed by CTCAE and the evaluation of the EORTC QLQ-CIPN20 and FACT-Taxane questionnaire across different treatment cycles. At cycle I, patients with G0 neurotoxicity reported significantly better scores compared to those with grade 1 toxicity. This trend was consistent in cycles IV and XII, where higher toxicity grades correlated with worse scores on both questionnaires. The assessment of QOL using FACT-Taxane showed deteriorating scores over time, with significant variances between baseline and subsequent cycles. Still, no significant difference was observed between cycles IV and XII.

Our analysis revealed a significant correlation between the EORTC QLQ-CIPN20 and the FACT-Taxane, specifically for the Tax-Subscale, indicating their similarity in assessing neurotoxicity.

Our results confirm those yet available in literature in other cancer types and disease settings. However, the literature on taxane-induced neuropathy in non-metastatic breast cancer is limited. Research suggests that combining the EORTC QLQ-CIPN20 with traditional physician-based clinical rating scales offers a more comprehensive understanding of the nature, frequency, and severity of CIPN in different oncology patient populations [8].

Although CIPN is a significant side effect induced by taxanes, there is no standardized tool for its evaluation. Most trials rely on the CTCAE, but this method presents limitations due to observer variability and data interpretation [20].

To assess the impact of CIPN on QOL, the EORTC-QLQC30 questionnaire is commonly used. However, other assessment scales, such as the FACT/GOG-Ntx, EORTC QLQ-CIPN20, and Total Neuropathy Score, have been developed to provide a more precise and reliable description of symptoms even though they are not yet standard measures [8].

In this study, the use of the mentioned questionnaires enabled us to directly assess the relationship between QOL and CIPN by focusing on symptoms. In contrast, existing literature typically reports a reduction in QOL indirectly, often through psychological stress evaluation. While our study did not measure psychological factors such as distress, depression, or anxiety, other research on TIN suggests that a decline in quality of life is often associated with persistent psychological distress, which can worsen the perception and severity of TIN-related symptoms [21].

Our research investigated the onset of TIN and its impact on QOL, demonstrating its early effect on patients’ well-being. This study highlights the effectiveness of innovative and highly sensitive tools for evaluating TIN through patient-reported outcome measures, specifically the EORTC QLQ-CIPN20 and the FACT-Taxane questionnaire. These tools were compared to conventional clinical strategies used in clinical practice, like CTCAE.

Our findings demonstrate that paclitaxel-induced neurotoxicity significantly impairs QOL in patients, even at relatively low cumulative doses. This impact becomes evident as early as 1 week after the fourth weekly dose and persists by the twelfth weekly dose, suggesting an ongoing effect in the short term. However, the literature offers limited insights into the short-term evaluation of TIN and its immediate effect on QOL.

A recent review by Schwab et al. examined the long-term impact of TIN on QOL in breast cancer survivors, finding that only four studies addressed QOL measures related to TIN. These studies, including Eckhoff et al. [22], primarily focused on the persistent nature of TIN, with 15% of survivors experiencing long-lasting symptoms that continued to affect their QOL negatively, even 5 years after treatment [23]. Engvall et al. found that TIN in early-stage breast cancer survivors significantly reduced global QOL, with more severe TIN leading to greater impairments in functionality, personal finances, and overall well-being [24]. This scarcity of short-term evaluations highlights a gap in current research, underscoring the need for studies like ours to better understand TIN’s early effects on patients’ well-being.

On the other hand, some studies have suggested that CIPN in breast cancer survivors may not significantly affect QOL 1 year after treatment, likely due to the reduction and improvement of CIPN symptoms over time [25].

This highlights a limitation in our current study, as the short follow-up period may not fully capture long-term symptom changes. Moving forward, we aim to extend the follow-up period and continue improving our research to better understand the long-term impact of CIPN on QOL.

Our results demonstrate that the EORTC QLQ-CIPN20 and the FACT-Taxane questionnaire effectively capture the severity of neurotoxicity and its impact on daily functioning and overall well-being, even on short-term evaluation. The EORTC QLQ-CIPN20 provides detailed insights into specific neurotoxic symptoms and their impact, while the FACT-Taxane questionnaire focuses on symptoms related to taxane chemotherapy. Unlike the CTCAE, which mainly categorizes symptoms clinically, these tools provide a more patient-centered perspective, offering valuable insights into how neurotoxicity affects overall quality of life and enabling more tailored management strategies.

At the moment, we do not have effective therapeutic approaches specifically aimed at preventing paclitaxel-induced neurotoxicity. Therefore, in clinical practice, dose reduction or delayed administration is the most commonly applied strategy to alleviate neurotoxicity symptoms. In cases of persistent and debilitating neuropathy, treatment should be stopped, reducing the benefit in terms of disease control.

A more accurate and timely identification of these symptoms through specific patient-centered instruments, such as the tools used in the present study, during adjuvant treatment could enhance the application of potential preventive strategies. In particular, duloxetine significantly mitigates neurotoxicity, especially with oxaliplatin treatment [25, 26]. ASCO advises the use of tricyclic antidepressants, gabapentin, and a baclofen-ketamine topical gel [27]. Conversely, N-acetylcysteine and vitamin E offer limited benefits, while physical exercise may alleviate the symptoms [28, 29]. However, the American Society of Clinical Oncology does not recommend any specific drug for CIPN prevention. In cases with a favorable prognosis, early identification of the risk of persistent TIN, which can significantly impair QOL—particularly in older patients with significant comorbidities like diabetes—might necessitate adjustments in treatment planning. These adjustments could include holding or discontinuing taxane in the adjuvant chemotherapy regimen. Naturally, such decisions require careful communication about the benefits and risks for each patient.

## Conclusion

The EORTC QLQ-CIPN20 and the FACT-Taxane questionnaire are crucial for evaluating chemotherapy-induced neurotoxicity and its effects on quality of life. Together, these questionnaires demonstrate that increased neurotoxicity is associated with declining quality of life. Despite being time-consuming, integrating these tools into clinical practice may offer a comprehensive understanding of patients’ experiences, facilitating better management and improving patient outcomes. This approach, potentially combined with neuroprotective strategies addressing early and subclinical neurological damage, could prevent the onset of clinically evident CIPN, which is responsible for an irreversible compromise in the QOL of breast cancer survivors.

Further studies are warranted to identify practical neuroprotection approaches, their impact on TIN, and their effects on patients’ QOL.

## Data Availability

No datasets were generated or analysed during the current study.
